# The roles and therapeutic potentialof mesenchymal stem/stromal cells and their extracellular vesicles in tendinopathies

**DOI:** 10.3389/fbioe.2023.1040762

**Published:** 2023-01-19

**Authors:** Daniel Quintero, Carlotta Perucca Orfei, Lee D. Kaplan, Laura de Girolamo, Thomas M. Best, Dimitrios Kouroupis

**Affiliations:** ^1^ Department of Orthopaedics, UHealth Sports Medicine Institute, Miller School of Medicine, University of Miami, Miami, FL, United States; ^2^ Laboratorio di Biotecnologie Applicate all’Ortopedia, IRCCS Istituto Ortopedico Galeazzi, Milan, Italy; ^3^ Diabetes Research Institute & Cell Transplant Center, Miller School of Medicine, University of Miami, Miami, FL, United States

**Keywords:** tendinopathies, tendon, mesenchymal stem/stromal cells, cell-based therapy, extracellular vesicles, cell-free therapy

## Abstract

Tendinopathies encompass a highly prevalent, multi-faceted spectrum of disorders, characterized by activity-related pain, compromised function, and propensity for an extended absence from sport and the workplace. The pathophysiology of tendinopathy continues to evolve. For decades, it has been related primarily to repetitive overload trauma but more recently, the onset of tendinopathy has been attributed to the tissue’s failed attempt to heal after subclinical inflammatory and immune challenges (failed healing model). Conventional tendinopathy management produces only short-term symptomatic relief and often results in incomplete repair or healing leading to compromised tendon function. For this reason, there has been increased effort to develop therapeutics to overcome the tissue’s failed healing response by targeting the cellular metaplasia and pro-inflammatory extra-cellular environment. On this basis, stem cell-based therapies have been proposed as an alternative therapeutic approach designed to modify the course of the various tendon pathologies. Mesenchymal stem/stromal cells (MSCs) are multipotent stem cells often referred to as “medicinal signaling cells” due to their immunomodulatory and anti-inflammatory properties that can produce a pro-regenerative microenvironment in pathological tendons. However, the adoption of MSCs into clinical practice has been limited by FDA regulations and perceived risk of adverse events upon infusion *in vivo*. The introduction of cell-free approaches, such as the extracellular vesicles of MSCs, has encouraged new perspectives for the treatment of tendinopathies, showing promising short-term results. In this article, we review the most recent advances in MSC-based and MSC-derived therapies for tendinopathies. Preclinical and clinical studies are included with comment on future directions of this rapidly developing therapeutic modality, including the importance of understanding tissue loading and its relationship to any treatment regimen.

## 1 Tendon Biology under normal physiological conditions

The function of tendons is to effectively transmit a tensile load, generate elastic contractile energy, and offer additional support and structure, particularly for large muscle-tendon units. Under physiological conditions, tendons are composed of a dense fibrillar ECM of organized type I collagen, with smaller amounts of collagens III, V, XI, XII and XIV (Maffulli et al., 2000; Riley, 2004; Sharma and Maffulli, 2005). Proteoglycans and glycoproteins constitute the majority of non-collagenous matrix components and are believed to serve in assembly of collagen fibrils, tendon integrity, and fascicle sliding and recoiling (Yoon and Halper, 2005; Dunkman et al., 2014; Screen et al., 2015). Overall, connective tissue layers, namely the paratenon, epitenon and endotenon, surround the fiber bundles facilitating frictionless movement and supply of blood vessels, nerves, and lymphatics to deeper tendon structures (Sharma and Maffulli, 2006; Docheva et al., 2015; Lipman et al., 2018).

The abundance of a highly organized ECM comes at the expense of reduced cell number. Tendon structural integrity and functionality mainly relies on a regulated interplay among tendon cell subpopulations and a highly organized ECM. The two main subpopulations of cells are the tenocytes and tendon stem/progenitor cells (TSPCs). Tenocytes have been described as highly specialized, elongated, mechanosensitive fibroblastic-like cells that are responsible for both cellular metabolism and appropriate turnover of extracellular collagen. The second, less abundant population, includes more rounded cells with clonogenic, self-renewing and multipotent capabilities, specific transcriptional profiles, and tendon-related gene expression levels (Bi et al., 2007; Citeroni et al., 2020; Perucca Orfei et al., 2021). In several studies they were capable of forming tendon like tissue when transplanted *in vivo* (Rui et al., 2010; Zhang and Wang, 2010; Lovati et al., 2011; Lui, 2013). *In vitro* studies demonstrate that TSPCs exhibit tissue specific characteristics including increased tenocyte differentiation potential and a greater proliferation rate when compared to bone marrow-derived MSCs (BMSCs) (Tan et al., 2012). TSPCs share several surface antigen receptors with MSCs supporting the theory of a shared progenitor cell. The CD44^+^, CD90^+^, CD105^+^, CD146^+^, CD31^−^, CD45^−^ immunophenotypic profile is present in both TSPCs and MSCs derived from various tissue types (Stanco et al., 2014; Perucca Orfei et al., 2021). Of all these cell surface markers, CD146 is of particular importance. Studies showed that CD146^+^ MSCs constitute the *bona fide* perivascular component of the bone marrow and were shown to have a perivascular topography [reviewed in (Kouroupis, 2019a)]. We recently showed that CD146 expression is associated with innately higher immunomodulatory and secretory capacity, and thus potential therapeutic potency (Bowles et al., 2020). The CD146^+^ TSPC subpopulation is of particular importance as studies have demonstrated that CD146^+^ TSPCs delineate an interfascicular cell subpopulation that is recruited in tendon injury *via* its ligand laminin-α4 to promote endogenous tendon regeneration (Lee et al., 2015; Marr et al., 2021). Unlike most MSCs, TSPCs are Nestin^+^, a marker of undifferentiated neuroepithelial or muscular cells actively involved in cellular remodeling (Bernal and Arranz, 2018). Single-cell molecular profiling has correlated a Nestin^+^ TSPCs subpopulation with superior tenogenic potential compared to TSPCs whole population and therefore increased endogenous tendon repair capacity (Yin et al., 2016). Overall, it is believed that *via* differentiation into tenocytes and paracrine secretion of immunomodulatory mediators (cytokines, chemokines, exosomes, microRNAs), resident TSPCs assist in maintaining local tendon homeostasis and healing (Millar et al., 2021).

## 2 Macroscopic and microscopic aspects of tendinopathies

In the late 1990s, a distinction in the macroscopic findings of tendinosis and tendinitis was proposed together with a subsequent shift in the clinical terminology to separate tendinitis from tendinosis and tendinopathy (Khan et al., 2000; Maffulli et al., 2000; Sharma and Maffulli, 2006). Chronic tendon injury, appropriately named tendinopathy, is not exclusively an inflammatory process. Microscopic changes that are clinically silent may include mucoid degeneration of the extracellular matrix (ECM). This is accompanied by cellular metaplasia with an increased concentration of cytokines, chemokines, and pro-inflammatory mediators (Praveen Kumar et al., 2019). Impaired tissue repair in the absence of rupture therefore represents a fundamental paradigm shift in our understanding of tendinopathy pathophysiology and subsequent treatment.

Currently, tendinopathies are defined as a condition of tendon non-healing where chronic dysregulation of local homeostasis is established. Fu and others proposed a unified theory for tendinopathy that follows a three-stage process (Fu et al., 2010). Firstly, the pathology is initiated by an unfavorable mechanical microenvironment which produces a proinflammatory injury. Second, there is diversion of the normal healing process which leads to a protracted course of local inflammation and oxidative stress. Lastly, there is an abundance of collagenolytic damage co-existing with impaired healing which can also contribute to patient symptoms. The exact mechanisms behind the abnormal early inflammatory response following tendon injury are not fully understood. However, emerging evidence suggests that alarmins released from necrotic cells constitute important triggers for the ensuing inflammatory response. During the early stages of tendon micro trauma (within 2 weeks from injury), changes of extracellular tissue microenvironment and activation of the innate immune system interact at a crossroads between reparative versus degenerative “inflammatory” healing. Specifically, following the initial tendon insult, resident immune-sensing cells react rapidly as sentinels through damage-associated molecular patterns (DAMPs) and together with an aberrant activation of tenocytes contribute to the recruitment of infiltrating immune cells (T cells, mast cells, and macrophages). Endogenous agents produced by tenocytes and infiltrating immune cells provoke inflammation due to the activation of inflammatory mediator pathways (TNF-α, IL-1β, IL-6, IL-8) and prostaglandins (PGE2) which promote pro-inflammatory macrophage (M1) and T cell activity (IL-17α releasing T cells). When the concentration of immune cells reaches a critical point, the massive release of inflammatory cytokines affects the reparative-degenerative homeostatic balance. Additionally, substance P (SP) neuropeptide can be actively secreted by peripheral sensory neurons and tenocytes generating neurogenic inflammation, pain, edema, and fibrosis in tendinopathy. Upon secretion, SP binds to neurokinin 1 receptors of mast cells, causing them to degranulate and release histamine, and also stimulating the *de novo* synthesis of leukotriene and prostaglandin (Scott and Bahr, 2009; Backman et al., 2011; Pingel et al., 2013). Lastly, increased matrix metalloprotease (MMP1 and MMP7) release promotes degradation of tendon ECM and may lead to persistence of tendinopathies, which is considered as a protracted, dysregulated, and maladaptive response to injury (D'Addona et al., 2017; Millar et al., 2017; Russo et al., 2022a).

**FIGURE 1:**
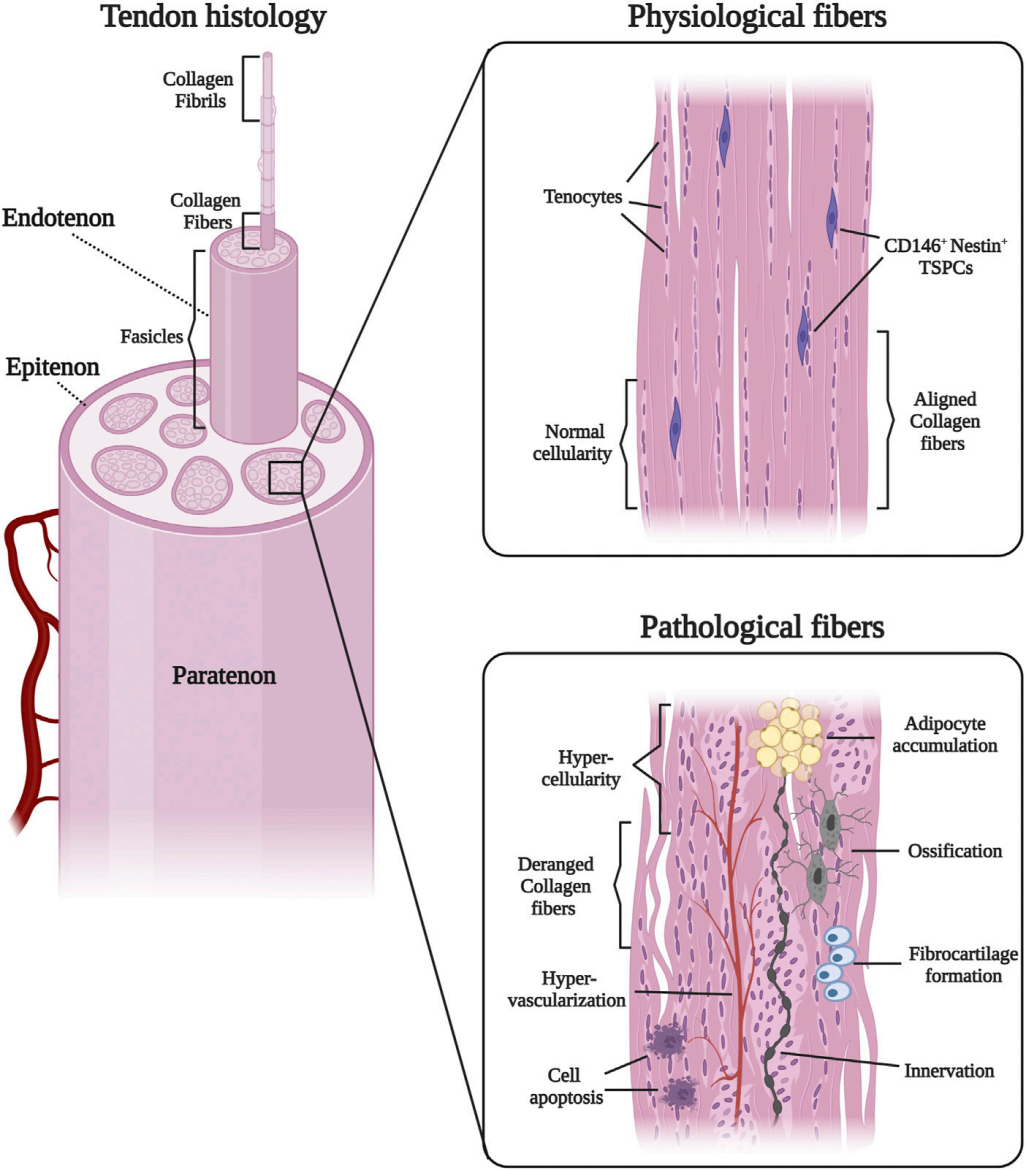
General tendon histology, and features of physiological and pathological tendon.

Physiologically, in these inflammatory conditions TSPCs can intervene, differentiate into tenocytes, and secrete appropriate ECM. Under pathological conditions, chronic exposure to high levels of inflammatory cytokines, growth factors, and proteases specific to the senescence associated secretory phenotype (SASP) profile, lead TSPCs to undergo metaplasia and to produce inflammatory mediators that amplify thermal and hypoxic stress (Millar et al., 2013; Docheva et al., 2015). In pathological conditions, TSPCs can differentiate towards non-tendon lineages including fat, bone, and cartilage cells, and therefore may contribute to heterotopic ossification and fatty infiltration in the tendon (Bi et al., 2007; Hu et al., 2016). Additionally, pathological human tendons show alterations in the total concentration of matrix metalloproteases, collagen I, and collagen III among other extracellular components (Rees et al., 2014) (Figure 1). At its core, the pathophysiology of chronic tendinopathy is an inability of tendon cellular regeneration, which leads to greater susceptibility, to repetitive microtrauma, and poor tissue repair.

## 3 The rationale for new cell-based therapeutics and the promise of mesenchymal stem cell-based approaches

As the etiology of tendinopathy has been attributed for years to be inflammatory (Cook et al., 2016), its conservative treatment have relied on the use of NSAIDs as the first choice. Still today oral and topical non-steroidal anti-inflammatory drugs (NSAIDs) are considered a reasonable treatment to minimize pain and restore normal function given their wide accessibility (Andres and Murrell, 2008), albeit numerous studies demonstrated their limited efficacy in treating tendinopathy beyond short-term pain relief (Bussin et al., 2021). Usually, up to 75% of individuals are responsive to conservative therapies that include activity level reduction, with or without immobilization together with a course of physical therapy usually emphasizing eccentric loading of the affected muscle-tendon complex (Stasinopoulos, 2015).

Considering etiology and microscopic findings in patients with tendinopathies, it is understandable that applications based on the use of mesenchymal stem cells (MSCs) have captured interest. Firstly identified by Friedenstein and others as multipotent cells within the bone marrow (Friedenstein et al., 1974)*,* a substantial more up-to-date literature supports the notion that most, if not all, MSCs are derived from the differentiation of perivascular or mural cells, so called pericytes, which are responsible for vascular maintenance and repair (Caplan, 2017). The healing potential for MSCs was further expanded following the discovery that they exert their regenerative effects not exclusively by cell-to-cell contact but also by their immunomodulatory/trophic paracrine activity *via* secretion of various cytokines, chemokines, exosomes, and microRNA (Caplan and Correa, 2011). Of these two modes of action most of the literature over the past decade suggests that the paracrine mechanism plays a role in most of the therapeutic outcomes. Furthermore, this paracrine response is theorized to be the prevailing mechanism of action of MSCs and which is to be exploited to treat a number of pathologies of the musculoskeletal system, including tendinopathies (Rui et al., 2013).

### 3.1 MSC-based approaches: Immunomodulatory actions of MSCs in preclinical models

Rather than direct participation in tendon healing/regeneration, the role of MSCs in the treatment of tendinopathies seems to be related to anti-inflammatory and analgesic properties that target local tissue pathophysiology and provide an extracellular environment conductive to proper tissue regeneration (Caplan and Correa, 2011; Kouroupis et al., 2019b; Bowles et al., 2020). MSCs derived from bone marrow, adipose tissue and umbilical cord are the most studied in animal models of tendinopathy. Several studies have demonstrated the strong immunomodulatory effects of MSCs as upon infusion they reduce total mononuclear cell infiltration and promote a regenerative/anti-inflammatory M2 macrophage phenotype in healing tendons (Table 1). Specifically, MSCs infused into tendons show a significant increase at the mRNA and/or protein levels for CD163, MRC1, and CD204 M2 macrophage markers, as well as IL-2, IL-4, prostaglandin reductase-1, and VEGF compared to control tendons (Gelberman et al., 2016; Shen et al., 2016; Yuksel et al., 2016; Gelberman et al., 2017; Shen et al., 2018). In addition, combined infusion of MSCs with tenogenic factors (BMP-12 or CTGF) results in more enhanced expression of IL-4 and decreased expression of pro-inflammatory mediators IL-1β, IL-6, and IFN-γ (Gelberman et al., 2017; Shen et al., 2018). Furthermore, preclinical studies have shown that *a priori* MSC functionalization *in vitro via* cell priming can boost their immunomodulatory capacity *in vivo* [reviewed in (Kouroupis et al., 2019a)]. Aktas et al. studied the *in vivo* healing effects of TNF-α primed MSCs compared to naïve MSCs in a rat Achilles segmental defect model. The authors reported that regardless of priming, MSCs increased IL-10 production and reduced IL-1α. However, primed MSCs additionally reduced IL-12 production and the number of M1 macrophages, as well as increased M2 macrophages, and the anti-inflammatory factor IL-4 (Aktas et al., 2017). Collectively, these studies demonstrate that macrophages and their M2 polarization driven by MSC immunomodulatory actions play a particularly important role in tendinopathy resolution.

**TABLE 1:** Immunomodulatory and reparative actions of MSCs in preclinical tendinopathy models.

References	Tendon injury	Number, age, and gender of animals	Therapeutic group	Control group	Follow-up	Therapeutic effect	Outcomes
Awad et al. (1999)	Surgically-induced patellar tendinopathy rabbit model	Eighteen 12 months-old female rabbits	Autologous BMSCs suspended in collagen I gel (5 × 10^6^ cells/mL)	Collagen I gel	4 weeks	Benefit	Compared to their matched controls, the MSC-mediated repair tissue demonstrated significant increases of 26% (*p* < .001), 18% (*p* < .01), and 33% (*p* < .02) in maximum stress, modulus, and strain energy density, respectively
							Improved mechanical properties, increased numbers of mature collagen fibers, minor increase in tenocyte number, compared to untreated controls
Chong et al. (2007)	Surgically-induced Achilles tendinopathy rabbit model	Fifty-seven 7.5 ± 1.24months-old female rabbits	Autologous BMSCs in fibrin carrier	Fibrin glue	3 weeks	Benefit	Early-stage improvement in tendon histological properties including increased collagen I possessing greater linear organization (*p* < .05) and biomechanical parameters (*p* < .05) compared to control untreated specimens
Crovace et al. (2007)	Healing secondary to overuse microtrauma tendon equine model	Three 4 years-old male Standardbred horses	Autologous BMSCs (5.5 × 10^6^ cells)	Saline	21 weeks	Benefit	BMSCs demonstrated improved histological scores (type lesion score, fiber pattern score and percentage of cross sectional area of the lesion) when compared to saline injected controls (*p* < .05)
Lacitignola et al. (2008)	Suspensory ligament or superficial digital flexor tendon equine model	Six adult Standardbred horses	Autologous BMSCs (5.5 × 10^6^ cells)	Saline or fibrin	21 weeks	Benefit	Sixty percent of the treated horses returned to sporting activity along with a decrease in lameness in 100% of the animals, and a reduced rate of re-injury compared to the non-treated group
Gulotta et al. (2009)	Unilateral detachment and repair supraspinatus tendon rat model	Ninety-eight mature male rats	Allogeneic BMSCs in fibrin glue (10^6^ cells)	Fibrin glue	4 weeks	Limited Benefit	Histological analysis to determine fibrocartilage deposition and conduction of biomechanical testing revealed no differences between allogeneic BMSC injected and control groups
Gulotta et al. (2011)	Unilateral detachment and repair supraspinatus tendon rat model	Sixty mature rats	Allogeneic BMSCs in fibrin glue (10^6^ cells)	N/A	4 weeks	Limited Benefit (for non-transduced BMSCs)	BMSC injected group had lower tensile strength, decreased load to failure and decreased tendon stiffness when compared to an Ad-Scx (adenoviral mediated scleraxis) induced BMSC injected group
			Allogeneic adenoviral-mediated scleraxis transduced BMSCs (10^6^ cells)				Ad-Scx group had more fibrocartilage (728.7 ± 50.4 vs. 342.6 ± 217.0 mm^2^; *p* = 0.04), higher ultimate load to failure (26.7 ± 4.6 vs. 20.8 ± 4.4 N; *p* = .01), higher ultimate stress to failure (4.7 ± 1.3 vs. 3.5 ± 1.0 MPa; *p* < .04), and higher stiffness values (15.3 ± 3.4 vs. 9.3 ± 2.2 N/mm; *p* < .001) as compared with the BMSC injected group
Uysal and Mizuno, (2011)	Achilles tendinopathy rabbit model	Ten 4 weeks-old rabbits	Allogeneic ASCs with platelet rich plasma (1.5 × 10^7^ cells)	N/A	4 weeks	Benefit	ASC-injected group showed significant increases in tensile strength together with increased levels of collagen I, FGF, VEGF compared to a platelet rich plasma alone injected control group
Lange-Consiglio et al. (2013b)	Acute tendon injury equine model	Ninety-two adult horses	Allogeneic AMSCs (5 × 10^6^ cells)	N/A	48 weeks	Benefit	No significant adverse effects after MSCs treatment
			Autologous BMSCs (5 × 10^6^ cells)				Animals belonging to AMSCs-infusion resumed their activities earlier (4–54months) compared to BMSCs-infusion group (4–12 months) after treatment
							The rate of re-injury in horses treated with AMSCs was lower (4.00%) compared with the average observed when horses were treated with BMSCs (23.08%)
Martinello et al. (2013)	Enzymatically-induced tendinopathy sheep model	Eighteen female sheep	Autologous PBMSCs (10 × 10^6^ cells)	Platelet rich plasma	17 weeks	Benefit	Significant differences were found between treated and control groups in tendon morphology and ECM composition
			Autologous PBMSCs with platelet rich plasma (10 × 10^6^ cells)				The combined use of PBMSCs and platelet rich plasma did not produce an additive or synergistic regenerative response
Smith et al. (2013)	Superficial digital flexor tendon injury equine model	Thirteen 7.8 ± 3.0 years-old male horses	Autologous BMSCs (10 × 10^6^ cells)	Saline	24 weeks	Benefit	BMSCs provides significant benefits compared to untreated tendon repair in enhancing normalization of biomechanical, morphological, and compositional parameters
							- Treated tendons had lower structural stiffness (*p* < .05) although no significant difference in calculated modulus of elasticity, lower (improved) histological scoring of organisation (*p* < .003) and crimp pattern (*p* < .05), lower cellularity (*p* < .007), DNA content (*p* < .05), vascularity (*p* < .03), water content (*p* < 0.05), GAG content (*p* < .05), and MMP-13 activity (*p* <.02)
Adams et al. (2014)	Achilles tendon transection rat model	Fifty-four 96 ± 22 days-old male rats	Human BMSCs with sutures (1 × 10^6^ cells)	Sutures	4 weeks	Benefit	BMSCs infusion resulted in tendon healing improvements *via* superior tissue biomechanical properties compared to control groups
							Histology score in the BMSCs infusion group was significantly lower (better) than in control group (*p* ≤ 0.001)
Behfar et al. (2014)	Surgically-induced deep digital flexor tendon injury rabbit model	Forty-eight adult male rabbits	Allogeneic stromal vascular fraction (SVF) from adipose tissue (4 × 10^6^ cells)	Saline	8 weeks	Benefit	SVF infusion group showed superior long-term biomechanical properties compared to the BMSC group
			Allogeneic BMSCs (4 × 10^6^ cells)				SVF treated tendons showed higher degrees of energy absorption, stress and stiffness compared to the BMSCs treated group (*p* < .05)
Deng et al. (2014)	Surgically-induced Achilles tendinopathy rabbit model	Thirty 4 weeks-old rabbits	Autologous ASCs seeded on PGA/PLA fibers (5 × 10^7^ cell/mL)	Cell-free scaffold	45 weeks	Benefit	ASC PLA/PGA constructs resulted in neo-tendon formation with histological structure and biomechanical properties similar to that of native tendon
							There were significant differences in both collagen fibril diameter and tensile strength between treated and control groups (*p* < 0.05)
Machova Urdzikova et al. (2014)	Collagenase-induced Achilles tendinopathy rat model	Eighty-one adult rats	Allogeneic BMSCs (1 × 10^6^ cells)	Saline	6 weeks	Benefit	BMSCs injected group demonstrated superior tendon ECM structure and larger amount of collagen I and collagen III deposition compared to the control group
							Neovascularization was also increased upon BMSCs infusion
Selek et al. (2014)	Achilles tendon transection rat model	Forty female rats	Allogeneic BMSCs (1 × 10^6^ cells)	Saline	12 weeks	Benefit	BMSCs infusion resulted in tendon healing improvements *via* anti-apoptotic effects and superior tissue biomechanical properties compared to control groups
							Increase in tendon strength was significantly higher in the study group than in the control group (*p* < .05)
Chen et al. (2015)	Collagenase-induced rotator cuff injury rat model	One hundred- twenty 12.5 weeks-old female rats	Human ASCs (3 × 10^8^ cells/mL)	Saline	4 weeks	Benefit	ASCs infusion restore the tensile strength and attenuate the progression of tendinitis
							The load to failure of the ASCs-injected group (15.87 ± 2.20 N) was notably higher than that of the saline-injected group (11.20 ± 1.35 N)
Chiou et al. (2015)	Achilles tendon midsubstance full-thickness defect rat model	Fifty-five rats	Allogeneic ASCs (2 × 10^6^ ASCs/mL)	Saline or tendon hydrogel or tendon hydrogel and platelet rich plasma	8 weeks	Benefit	All groups demonstrated increased strength, cellularity, and ECM formation compared to control groups
			Allogeneic ASCs with platelet rich plasma (2 × 10^6^ ASCs/mL)				Hydrogel with platelet rich plasma and ASCs demonstrated increased strength over other groups (*p* < 0.05)
			Allogeneic ASCs with platelet rich plasma and biocompatible tendon hydrogel (2 × 10^6^ ASCs/mL)				
He et al. (2015)	Flexor tendon healing rabbit model	Female rabbits	Autologous BMSCs with fibrin glue (1 × 10^6^ cells)	Fibrin glue	3 weeks	Benefit	BMSCs infusion resulted in increased range of motion but without improvement of biomechanical properties compared to a control group
			Allogeneic BMSCs with fibrin glue (1 × 10^6^ cells)				
			Allogeneic BMSCs with fibrin glue (4 × 10^6^ cells)				
Gelberman et al. (2016)	Flexor tendon repair canine model	Seventeen adult dogs	Autologous ASCs with BMP-12 seeded on heparin/fibrin-based scaffold	Scaffold	4 weeks	Limited Benefit	No effect of ASCs with BMP12 treatment for range of motion or tensile properties outcomes versus repair only
							Treatment with ASCs with BMP12 amplified inflammation, stress response, and matrix degradation
Shen et al. (2016)	Flexor tendon repair canine model	Twelve female dogs	Autologous ASCs sheets with thiol-modified hyaluronan hydrogel	Hyaluronan hydrogel	1 week	Benefit	ASCs promoted a regenerative/anti-inflammatory M2 macrophage phenotype and regulated tendon matrix remodeling
							Significant increases in M2-stimulator (IL-4), marker (CD163 and MRC1), and effector (VEGF) gene expression in ASCs treated tendons compared with non-treated tendons
Yuksel et al. (2016)	Surgically-induced Achilles tendinopathy rat model	Thirty-five 12 months-old male rats	Allogeneic BMSCs (1 × 10^6^ cells)	Saline or platelet rich plasma	4 weeks	Benefit	Levels of pro-inflammatory cytokines TNF-α, IFNγ, and IL 1β were significantly low in BMSCs group (*p* < 0.05)
							Levels of anti-inflammatory cytokines and growth factors, such as IL2, VEGF, TGF-b, and HGF, were significantly higher in the MSC group than those of the platelet rich plasma and control groups (*p* < .05)
Aktas et al. (2017)	Achilles segmental defect rat model	Seventy-two mature male rats	Allogeneic BMSCs with poly(lactide-co-glycolide) scaffold (1 × 10^6^ cells)	Poly(lactide-co-glycolide) scaffold	4 weeks	Benefit	Regardless of priming, BMSCs increased IL-10 production and reduced IL-1α
			Allogeneic TNF-a primed BMSCs with poly (lactide-co-glycolide) scaffold (1 × 10^6^ cells)				Primed BMSCs additionally reduced IL-12 production and the number of M1 macrophages, as well as increased M2 macrophages, and the anti-inflammatory factor IL-4
Geburek et al. (2017)	Surgically-induced superficial digital flexor tendon injury equine model	Nine 3–6 years-old horses	Autologous ASCs with inactivated autologous serum (10 × 10^6^)	Inactivated autologous serum	24 weeks	Limited Benefit	AMSCs implantation did not substantially influence clinical and ultrasonographic parameters
							Histology, biochemical and biomechanical characteristics of the repair tissue did not differ significantly between ASCs and control treatment modalities
							Compared with macroscopically normal tendon tissue, the content of the mature collagen crosslink hydroxylysylpyridinoline did not differ after ASCs-serum treatment (*p* = 4.074) while it was significantly lower (*p* = .027) in lesions treated with serum alone. Stress at failure (*p* = .048) and the modulus of elasticity (*p* = .001) were significantly lower after ASCs-serum treatment than in normal tendon tissue
Gelberman et al. (2017)	Flexor tendon repair canine model	Sixteen 1–2 years-old female dogs	Autologous ASCs sheets	Normal tendon	2 weeks	Benefit	Improved healing response together with increased tendon ECM regeneration compared to controls
			Autologous ASCs sheets with rBMP-12				ASCs sheet treatment modulated the post repair inflammatory response and facilitated healing by increasing regenerative M2 macrophages and CD146+ stem or progenitor cells
							ASCs with rBMP-12 further stimulated M2 macrophages by increasing IL-4 (116-fold of normal, *p* = .002) and led to the increase of M2 effector matrix metalloproteinase-12 involved in matrix remodeling (2-fold of normal, *p* = .016) and reduction of a negative regulator of angiogenesis and cell migration (StAR-related lipid transfer domain protein13; 84% of normal, *p* = .000)
Lee et al. (2017)	Achilles tendon full-thickness rectangular defect rat model	Fifty-seven 13 weeks-old male rats	Human ASCs suspended in fibrin glue (10^6^)	Fibrin glue	4 weeks	Benefit	ASCs suspended in fibrin glue resulted in better gross morphological and biomechanical recovery than the control group
							The expression of both collagen I and tenascin-C was significantly higher in the cell group (*p* = .011 and *p* = .327, respectively)
Peach et al. (2017)	Surgically-induced rotator cuff injury rat model	Sixty-six mature male rats	Allogeneic ASCs seeded on PCL/PNEA-mPh fiber matrices (3.0 × 10^4^ cells)	PCL/PNEA-mPh fiber matrices	12 weeks	Benefit	ASCs infusion accelerate the restoration of tendon tensile strength coupled with improved fiber alignment and tendon organization
Romero et al. (2017)	Surgically-induced superficial digital flexor tendon injury equine model	Twelve 5–8 years-old horses	Autologous BMSCs (20 × 10^6^ cells)	Saline	45 weeks	Benefit	Early stage (6 weeks) the ultrasound echogenicity score in tendons treated with BMSCs suggested earlier improvement, whilst all treatment groups reached the same level at week 10, which was superior to the control group
			Autologous ASCs (20 × 10^6^ cells)				Gene expression was indicative of better tissue regeneration after all treatments, especially for BMSCs, as suggested by upregulation of collagen type I, decorin, tenascin and matrix metalloproteinase III mRNA
			Platelet rich plasma				
Ahrberg-Spiegl et al. (2018)	Surgically-induced superficial digital flexor tendon injury equine model	Six horses 3–10 years-old horses (3 female, 3 male)	Autologous ASCs with autologous serum (10^7^ cells)	Autologous serum	24 weeks	Limited Benefit	Limited compositional or structural tendon improvement after MSC treatments
							Symptoms decreased in both ASC-treated and control groups
							In ASC-treated tendons, mean lesion signal intensity as seen in T2w magnetic resonance imaging and cellularity as seen in the histology (*p* < .05) were lower
Cai et al. (2018)	Surgically-induced Achilles tendinopathy rabbit model	Sixteen mature rabbits	Allogeneic BMSCs seeded on polyethylene terephthalate (PET) (2 × 10^7^ cells/mL)	PET	12 weeks	Benefit	BMSCs seeded PET group was superior histologically (increased collagen I and III expression, *p* = .002 and *p* = .006, respectively) compared to the control untreated group
							BMSCs seeded PET group was superior biomechanically (higher failure load and average stiffness) compared to the control untreated group
							The failure load in the BMSCs seeded PET group was higher than that in the PET group (124.5 ± 5.5 N vs. 107.8 ± 5.3 N; *p* = .021)
							The average stiffness in the BMSCs seeded PET group was significantly greater than that in the PET group (30.0 ± 2.4 N/mm vs. 22.8 ± 2.8 N/mm; *p* = .021)
de Aro et al. (2018)	Surgically-induced Achilles tendinopathy rat model	One hundred-ten 120 days-old male rats	Allogeneic ASCs (3.7 × 10^5^ cells)	Saline	2 weeks	Limited Benefit (for ASCs with GDF-5)	ASCs with GDF-5 group showed inferior tenogenic gene expression, hydroxyproline concentration, collagen fiber organization, and tendon biomechanics compared to ASCs only group
			Allogeneic ASCs with GDF-5 (3.7 × 10^5^ cells)				
Kwon et al. (2018)	Full thickness subscapularis tendon tear rabbit model	Twenty-four 12 weeks-old male rabbits	Human UCMSCs (1 × 10^6^ cells)	N/A	4 weeks	Benefit	Consistent improvement in gross morphology for all concentrations of UCMSCs injections, with 25% of all experimental animals demonstrating a full recovery
			Human UCMSCs with polydeoxyribonucleotide (1 × 10^6^ cells)				On histological assessment there was appropriate parallel arrangement of hypercellular fibroblastic bundles as well as an increased presence of collagen I fibers
Shen et al. (2018)	Flexor tendon repair canine model	10 adult female dogs	Autologous ASCs sheets with connective tissue growth factor (CTGF) (16,000 cells/cm^2^)	Non-treated	2 weeks	Benefit	Combining ASCs with CTGF reduced the inflammatory IFNG (*p* = .014) and scar-associated COL3A1 (*p* = .007) gene expression compared to control group and CTGF alone, respectively
			CTGF				Combining ASCs with CTGF yields increased expression of CD146+ TSPCs at the tendon surface and interior core during healing
Russo et al. (2022a)	Sheep Achilles tendon injury model	Forty 2 years-old male sheep	Allogeneic mesenchymal amniotic epithelial stem cells with firbin glue (mAECs, 10^7^ cells)	Fibrin glue	4 weeks	Limited Benefit (for mAECs)	eAECs and tdAECs had better significant histological scores with respect to mAEC-treated tendons
			Allogeneic amniotic epithelial stem cells with fibrin glue (eAECs, 10^7^ cells)				A greater COL1/COL3 ratio was recorded in all the typologies of allotransplanted tendons (control vs. mAECs, *p* < .01 and control vs. eAECs or tdAECs, for both *p* < .05)
			Allogeneic tendon-like amniotic epithelial stem cells with fibrin glue (tdAECs, 10^7^ cells)				The immunomodulatory response at day 14 showed in eAEC-transplanted tendons an upregulation of pro-regenerative M2 macrophages with respect to mAECs and tdAECs
							An overall higher IL10/IL12 characterized all subsets of AECs-treated tendons even if the more favorable ratio was recorded in eAECs-treated

### 3.2 MSC-based approaches: Reparative actions of MSCs in preclinical models

Restoration of tendon ECM molecular composition and architecture is a major goal in regenerative therapies. The innate paracrine trophic and reparative capacities of MSCs as mechanisms to facilitate local changes in various milieus are well known (Caplan and Correa, 2011; Salgado et al., 2018). In tendon healing, studies have shown that MSCs are capable of synthesizing a considerable amount of tendon-specific ECM [reviewed in (Burk, 2019)], however their therapeutic effect can also be exerted *via* stimulatory effects on tenocytes or TSPCs, which in turn are capable of synthesizing new tendon ECM (Table 1).

BMSCs have been tested for the treatment of Achilles, superficial digital flexor, patellar and rotator cuff tendon in rabbit, horse and rat animal models. In two separate rat Achilles tendon transection studies, human BMSCs or rat allogeneic BMSCs infusion resulted in tendon healing improvements *via* anti-apoptotic effects and superior tissue biomechanical properties compared to control groups (Adams et al., 2014; Selek et al., 2014). Autologous BMSCs suspended in collagen I gel were able to prompt tissue repair upon implantation in a surgically-induced patellar tendinopathy rabbit model, leading to improved mechanical properties as well as increased numbers of mature collagen fibers along with a minor increase in tenocyte number, compared to untreated controls at 4 weeks (Awad et al., 1999). Similar findings were reported in another study employing a surgically-induced Achilles tendinopathy rabbit model, showing that intra-tendinous infusion of autologous BMSCs was associated with early stage improvement in tendon histological properties including increased collagen I possessing greater linear organization and biomechanical parameters compared to control untreated specimens (Chong et al., 2007). In another study using a tissue engineering approach, polyethylene terephthalate (PET) seeded with allogeneic BMSCs cultured *vitro* was implanted for Achilles tendon repair in a surgical dissection rabbit model (Cai et al., 2018). Interestingly, the BMSCs seeded PET group was superior both histologically (increased collagen I and III expression), and biomechanically (higher failure load and average stiffness) compared to the control untreated group. Significant improvements in healing were observed in a collagenase-induced Achilles tendinopathy rat model after allogeneic human BMSCs local infusion, where the BMSC injected group demonstrated superior tendon ECM structure and larger amount of collagen I and collagen III deposition compared to the control group. Neovascularization was also increased upon BMSCs infusion (Machova Urdzikova et al., 2014). In a rabbit flexor tendon healing model, autologous BMSCs infusion resulted in increased range of motion but without improvement of biomechanical properties compared to a control group (He et al., 2015). A study investigating healing secondary to overuse microtrauma in an equine model reported animals receiving autologous BMSCs demonstrated improved histological scores when compared to saline injected controls (Crovace et al., 2007). A separate study by the same authors utilized autologous BMSCs and a standard course of physical therapy in 20 horses with suspensory ligament or superficial digital flexor tendon tendinopathy. Sixty percent of the treated horses returned to sporting activity along with a decrease in lameness in 100% of the animals, and a reduced rate of re-injury compared to the non-treated group (Lacitignola et al., 2008). Additional studies using the equine tendinopathy model have shown that MSCs infusion results not only in inhibition of collagen degradation but also enhanced secretion of ECM matrix remodeling enzymes. Specifically, autologous BMSCs infusion decreased MMP-13 activity and increased MMP-3 expression in healing tendons, at 6 months and 45 weeks, respectively (Smith et al., 2013; Romero et al., 2017). However, there are few studies utilizing BMSCs that do not produce the same encouraging results demonstrated above. Namely two studies by Gulotta et al. utilized a rat model employing unilateral detachment and repair of the supraspinatus tendon. Histological analysis to determine fibrocartilage deposition and conduction of biomechanical testing revealed no differences between allogeneic BMSC injected and control groups (Gulotta et al., 2009). In another study by Gulotta et al. the reparative capacity of naïve BMSCs was compared to genetically modified BMSCs in the same rat model. At 4 weeks the BMSC injected group had lower tensile strength, decreased load to failure and decreased tendon stiffness when compared to an Ad-Scx (adenoviral mediated scleraxis) induced BMSC injected group (Gulotta et al., 2011). However, differences observed in MSCs’ effectiveness for tendon healing could be attributed to the MSCs dosage administered and duration of tissue histology and biomechanics follow-up evaluation.

Adipose-derived MSCs (ASCs) have also been used in preclinical studies for the treatment of tendinopathies, albeit less frequently. Uysal et al. utilized ASCs with platelet rich plasma (PRP) in a rabbit model of Achilles tendinopathy and demonstrated that the ASC-injected group showed significant increases in tensile strength together with increased levels of collagen I, FGF, VEGF compared to a PRP alone injected control group (Uysal and Mizuno, 2011). In another study, ASCs were co-infused with biocompatible tendon hydrogel and PRP into a Achilles tendon midsubstance full-thickness defect rat model. Results demonstrated increased strength, cellularity, and ECM formation compared to control groups (Chiou et al., 2015). Similarly, Lee et al. using a full-thickness rectangular defect Achilles tendon rat model showed that the implantation of human ASCs suspended in fibrin glue resulted in better gross morphological and biomechanical recovery than the control group. Of particular interest, the expression of both collagen I and tenascin-C was significantly higher in the cell group (Lee et al., 2017). Few studies have combined ASCs with tenogenic factors and assessed their therapeutic capacity *in vivo*. In one study, allogeneic ASCs were infused with and without GDF-5 in a rat model of surgically induced Achilles tendinopathy. The ASCs with GDF-5 group showed inferior tenogenic gene expression, hydroxyproline concentration, collagen fiber organization, and tendon biomechanics compared to ASCs only group (de Aro et al., 2018). Therefore, although other literature demonstrated the beneficial effect of GDF-5 for the tendon healing process, de Aro et al. show that its application cannot improve the repair process of partial transected tendons (de Aro et al., 2018). In two separate studies, autologous ASCs sheet co-infusion with BMP-12 or CTGF in a canine flexor tendon repair model resulted in an improved healing response together with increased tendon ECM regeneration compared to controls (Gelberman et al., 2017; Shen et al., 2018). Of note, combining ASCs with CTGF yields increased expression of CD146^+^ TSPCs at the tendon surface and interior core during healing (Shen et al., 2018). Using a tissue engineering approach, allogeneic ASCs were initially cultured for 5 weeks on a polylactic acid (PLA)/polyglycolic acid (PGA) scaffold coupled with mechanical loading *in vitro*. The implantation of ASC PLA/PGA constructs in an Achilles tendon surgical dissection rabbit model resulted in neo-tendon formation with histological structure and biomechanical properties similar to that of native tendon (Deng et al., 2014). ASCs have been used to treat rotator cuff injury. Two separate studies have demonstrated that both human and rat ASCs infusion accelerate the restoration of tendon tensile strength coupled with improved fiber alignment and tendon organization (Chen et al., 2015; Peach et al., 2017). Importantly, two comparative studies evaluated ASCs and BMSCs therapeutic capacity in flexor tendon transection models. In one study, allogeneic stromal vascular fraction (SVF) or BMSCs were infused into surgically induced lesions of a rabbit deep digital flexor tendon. The SVF infusion group showed superior long-term biomechanical properties compared to the BMSCs group (Behfar et al., 2014). In another study, Romero et al. evaluated the therapeutic capacity of ASCs, BMSCs, and PRP in surgically induced lesions of the equine superficial digital flexor tendon. Upregulated collagen I, decorin, tenascin-C and matrix metalloproteinase III gene expression suggested tissue healing, especially for the BMSC treated group. Interestingly, all treatment groups showed superior tendon regeneration compared to untreated controls (Romero et al., 2017).

Umbilical cord derived MSCs (UCMSCs) have been evaluated in the treatment of chronic, full thickness rotator cuff tears, but not for chronic tendinopathy or partial tendon tears. Specifically, Kwon et al. utilized human UCMSCs with polydeoxyribonucleotide in a rabbit model of a full thickness subscapularis tendon tear to emulate chronic changes (Kwon et al., 2018). There was a consistent improvement in gross morphology for all concentrations of UCMSCs injections, with 25% of all experimental animals demonstrating a full recovery at 4 weeks. On histological assessment there was appropriate parallel arrangement of hypercellular fibroblastic bundles as well as an increased presence of collagen I fibers (Kwon et al., 2018). Amniotic membrane derived MSCs (AMSCs) have also been used in proof-of-concept preclinical studies for the treatment of tendon injuries (Russo et al., 2022b). In a comparative study, Lange-Consiglio et al. evaluated AMSCs and BMSCs infusion therapeutic capacity in acute tendon injury equine model (Lange-Consiglio et al., 2013b). Importantly, no significant adverse effects after MSCs treatment were seen whereas animals belonging to AMSCs-infusion resumed their activities earlier (4–5 months) compared to BMSCs-infusion group (4–12 months) after treatment. Of note, the rate of re-injury in horses treated with AMSCs was lower (4.00%) compared with the average observed when horses were treated with BMSCs (23.08%) (Lange-Consiglio et al., 2013b). In a separate study, intravenous infusion of autologous peripheral blood MSCs (PBMSCs) with and without PRP demonstrated improved histologic features when compared to controls in an enzymatically induced tendinopathy sheep model (Martinello et al., 2013). Specifically, significant differences were found between treated and control groups in tendon morphology and ECM composition. However, the combined use of PBMSCs and PRP did not produce an additive or synergistic regenerative response (Martinello et al., 2013).

In most *in vivo* studies, tendon ECM composition was improved following MSC treatment, evident mainly by an increased amount of collagen I, tenascin-C, and decorin protein synthesis. In conjunction with ECM synthesis, the therapeutic effect MSCs is perhaps attributable to active ECM remodeling and the involvement of synthesized ECM molecules to enhance collagen fibrillogenesis. However, studies in large animal models have shown limited compositional or structural tendon improvement 5 months after MSC treatments (Geburek et al., 2017; Ahrberg-Spiegl et al., 2018). Taken together, although less studied than other clinical conditions, the use of MSCs offers promise for the treatment of tendinopathy, to date preclinical studies have demonstrate both MSC safety and efficacy upon *in vivo* infusion.

### 3.3 MSC-based approaches: Clinical studies

In humans, studies evaluating the outcome of cell-based approaches for tendon pathologies are scarce (Table 2). In 2012 a single arm-controlled trial on patellar tendinopathy was performed in a sub-population of eight patients who were refractory to conservative management for at least 6 months. Autologous bone marrow mononuclear cells (BMNCs) were harvested from the iliac crest and subsequently infused under ultrasound guidance into the patellar tendon lesion. Outcome measures included the Tegner, international knee documentation committee (IKDC) and knee injury and osteoarthritic outcome score (KOOS) assessments. All eight demonstrated significant 5-year follow-up improvement in functional scores after local injection with 30,000 BMNCs (Pascual-Garrido et al., 2012). In a separate study, Connell et al. reported improvement in tendon appearance by ultrasound decreased tendon thickness. The authors reported improved functional scores [Patient-Rated Tennis Elbow Evaluation (PRTEE)] compared to controls after collagen-producing cells derived from skin fibroblasts were locally injected for refractory elbow epicondylitis. Of the 12 individuals enrolled, 11 demonstrated satisfaction with their treatment that was not previously achieved with conventional therapies (Connell et al., 2009). In a previous randomized controlled clinical trial, both PRP and SVF were safe, effective treatments for recalcitrant Achilles tendinopathy. However, comparing the two treatment groups, VAS, AOFAS and VISA-A scored significantly better at 15 and 30 days in the SVF in comparison to PRP group (*p* < .05) (Usuelli et al., 2018). Similar results were reported for a randomized controlled trial comparing physical therapy and BMNCs + PRP treatment. Twelve patients underwent ultrasound guided injection with BMNCs + PRP whereas a matched control population underwent physical therapy alone. Pain scores and functional outcomes as reported by ASES were improved in BMNCs + PRP group at 3 months follow up (Kim et al., 2018).

**TABLE 2:** Clinical studies using MSC-based therapy for tendinopathy.

References	Study type	Tendon injury	Inclusion criteria	Patients	Therapeutic group	Control group	Follow-up	Therapeutic effect	Outcomes
Connell et al. (2009)	Prospective clinical pilot study	Refractory elbow epicondylitis	Refractory common extensor origin tendinosis with 18.1 months (range 6–24 months) mean duration of symptoms	12 (29–48 years-old, 5 males and 7 females)	Collagen-producing cells derived from dermal fibroblasts (10 × 10^6^ cells)	N/A	6 months	Benefit	- All patients reported improved functional scores (Patient-Rated Tennis Elbow Evaluation (PRTEE)) compared to controls (*p* < .05)
									The healing response on ultrasonography showed median decrease in: number of tears, from 5 to 2; number of new vessels, from 3 to 1; and tendon thickness, from 4.35 to 4.2 (*p* < .05)
									Of the 12 individuals enrolled, 11 demonstrated satisfaction with their treatment that was not previously achieved with conventional therapies
Pascual-Garrido et al. (2012)	Single arm-controlled trial	Patellar tendinopathy	History of pain (more than 6 months), tenderness on patellar tendon palpation, and magnetic resonance imaging findings of degenerative changes	8 (14–35 years-old, gender not reported)	Autologous bone marrow mononuclear cells (BMNCs) (3 × 10^4^ cells)	N/A	5 years	Benefit	All 8 demonstrated significant 5-year follow-up improvement in functional scores [Tegner, international knee documentation committee (IKDC) and knee injury and osteoarthritic outcome score (KOOS) assessments] after local injection
									Statistically significant improvement (preoperative to postoperative) for the Tegner (2–8, *p* = .006), IKDC scores (36–69, *p* = .047), KOOS symptoms (44–71 *p* = .0086), KOOS ADL (63–90, *p* = .0086), KOOS sport (24–63 *p* = .0078)
Usuelli et al. (2018)	Randomized controlled trial	Non-insertional Achilles tendinopathy	Unilateral or bilateral chronic tendinopathy of the Achilles tendon recalcitrant to traditional conservative treatments including oral medication and physical modalities; symptoms lasting for at least 3 months; VAS (visual analogue scale) pain at the first visit >5	44 (18–55 years-old, gender not reported)	Stromal vascular fraction (SVF) (4 mL obtained from 50 mL subcutaneous adipose tissue)	N/A	6 months	Benefit	Comparing the two groups, VAS pain scale, the VISA-A, the AOFAS Ankle-Hindfoot Score scored significantly better at 15 and 30 days in the SVF in comparison to platelet rich plasma group (*p* < .05)
					Platelet rich plasma (4 mL)				At the following time points the scores were not significantly different between the two groups
									No correlation has been found between clinical and radiological findings.
Kim et al. (2018)	Randomized controlled trial	Rotator cuff tendon tear	Shoulder pain for more than 3 months and no improvement by conventional oral medication and physical modalities	24 (age and gender not reported)	Autologous bone marrow mononuclear cells (BMNCs) with platelet rich plasma (2 mL BMNCs obtained from 30 mL peripheral blood)	Patients doing physical therapy	3 months	Benefit	Initial visual analog scale (VAS) and functional outcomes as reported by American Shoulder Elbow Surgeon score (ASES) were improved in BMNCs + PRP group
									The change in the VAS differed between groups at 3 months (*p* = .039)
									The ASES scores in the BMAC-PRP group changed from 39.4 ± 13.0 to 74.1 ± 8.5 at 3 months while those in the control group changed from 45.9 ± 12.4 to 62.2 ± 12.2 at 3 months (*p* = .011)
Jo et al. (2018)	Open-label, single-left, dose-escalation trial	Rotator cuff tendon tear	Unilateral shoulder pain for more than 3 months of symptom duration, and partial-thickness rotator cuff tear identified with ultrasonography or magnetic resonance imaging	18 (>19 years-old, gender not reported)	Autologous ASCs (1.0 × 10^7^, 5.0 × 10^7^, and 1.0 × 10^8^ cells in 3 mL of saline)	N/A	6 months	Benefit	ASCs injection resulted in improvement in their shoulder pain and disability index (SPADI) by 80% and 77% in the midand high-dose groups, respectively
									Shoulder pain was significantly alleviated by 71% in the high-dose group
									Magnetic resonance imaging examination showed that volume of the bursal-side defect significantly decreased by 90%
									in the high-dose group
									Arthroscopic examination demonstrated that volume of the articular- and bursal-side defects decreased by 83% and 90% in the mid- and high-dose groups, respectively
Khoury et al. (2021)	Prospective longitudinal case series exploratory study	Chronic lateral elbow tendinopathy	Pain and disability for at least	19 (mean age 46.5 years-old, 11 males and 8 females)	Allogenic ASCs (1 × 10^6^-1 × 10^7^ cells)	N/A	12 months	Benefit	ASCs injection resulted in 79% improvement in Initial visual analog scale (VAS) pain score and decreased functional disability as reported by Mean quick Disabilities of the Arm, Shoulder and Hand (QuickDASH) compulsory score
			4 months and no improvement by conventional oral/injectable medication and physical modalities						

Similar results were reported in two separate studies using ASCs for tendinopathy. Tendon injection of autologous ASCs in 18 patients with rotator cuff disease resulted in 80% improvement in their shoulder pain and disability index (SPADI). Patients also demonstrated diagnostic evidence of tendon repair *via* both MRI and arthroscopic examination of bursal and articular rotator cuff defects (Jo et al., 2018). In another study, injection of allogenic ASCs (1 × 10^6^-1 × 10^7^) at the common extensor tendon for the treatment of lateral epicondylitis resulted in 79% improvement in pain and decreased functional disability as reported by QuickDASH compulsory score for 1 year follow-up (Khoury et al., 2021).

Although none of the studies mentioned above report serious adverse effects, one of the greatest barriers to MSC incorporation into clinical practice is the concern for complications related to malignant transformation and hypersensitivity reactions. To identify the extent of complications and incidence of such events in a heterogenous population, Centeno et al. conducted a multi-center analysis of 2,372 patients who received MSCs injection for a number of orthopedic conditions. There were 325 adverse events reported for 3,012 procedures with an average follow up of 2.2 years. The majority of adverse events were pain post-procedure (n = 93, 3.9% of the study population) and pain due to progressive degenerative joint disease (n = 90, 3.8% of the study population). In reference to malignant transformation, the study identified that the number of newly diagnosed neoplasms was lower in individuals administered MSCs compared to the general population (Centeno et al., 2016). Importantly, another study showed that compared to a more conventional treatment, corticosteroid injections, MSCs provide comparable post procedure pain control, but their administration is not associated with myopathy, osteonecrosis, or osteoporosis (Hart, 2011).

Overall, preclinical and clinical studies reveal that MSC-based therapeutic approaches may improve pain, function, radiological, and arthroscopic parameters in tendinopathy. However, large-scale randomized controlled trials are needed to confirm no adverse effects and most importantly long-term functional improvements. Specifically, long-term adverse effects related to immunologic reactions and possible risk of microvascular occlusion of autologous or allogeneic MSC treatment should be thoroughly investigated. Further studies will also clarify and standardize technical issues related to mode of MSC infusion, MSC dosage, and number of MSC infusions for better long-term functional improvements. On this basis, further large-scale and long-term studies will help to overcome current hurdles for MSC clinical translation in tendinopathies.

## 4 Overview of cell-free applications for the treatment of tendon pathologies: Extracellular vesicles from MSCs

Despite demonstrating promising clinical effects for various pathologies (Phinney and Prockop, 2007), there are still critical issues related to MSC therapeutic applicability such as their short-term survival at the target tissue site to ensure an effective and long-term therapeutic effect. Understanding that the activity of MSCs is mediated *in vivo* mainly through a paracrine activity was instrumental to proposing a series of studies and applications based on this particular property (Vizoso et al., 2017). Therefore, attention is now focused on MSC secretome and their mechanisms involved in paracrine activity. MSC secretome is defined as a set of soluble and non-soluble factors released by MSCs which are responsible for a plethora of cellular modulations and responses within the tissue microenvironment (Lui, 2013). Together with their main constituents such as cytokines/chemokines, EVs and the embedded molecules they carry represent a valid candidate as a cell-free approach for various clinical indications (Vizoso et al., 2017). In the context of tendinopathies, few preclinical studies investigate the efficacy of the MSC secretome (Lange-Consiglio et al., 2013a; Lange-Consiglio et al., 2016; Sevivas et al., 2017; Sun et al., 2019). However, recent studies show that the MSC secretome does indeed have an effect in promoting healing of tendons and ligaments by showing significantly better functional and biomechanical outcomes compared to control groups (Rhatomy et al., 2020).

Extracellular vesicles (EVs) contained in MCS secretome represent the most interesting therapeutic candidate. EVs are lipid bilayer-delimited particles that are released into all body fluids by most eukaryotic cells in both normal and pathophysiological conditions. They are classified as exosomes or microvesicles. Exosomes are smaller in size (40–120 nm) and of endocytic origin while microvesicles are larger (50–1,000 nm) and originate from the cell membrane (Lui, 2013). Both act as carriers of a series of protein, lipid and nucleic acid components that reflect the condition of the cell they are derived from. In this way, two cells, even if distant, can communicate and subsequently modulate the other’s functionality. Accordingly, circulation of EVs in body fluids can have a potential influence on tissue physiology, as they promote systemic reparative and anti-inflammatory mechanisms to restore healthy function in damaged cells (Gomez-Salazar et al., 2020).

To date, preclinical studies utilizing MSC-derived EVs have shown their ability to induce anti-inflammatory, anti-apoptotic and angiogenic responses, and to reprogram somatic cells towards a regenerative process (Kou et al., 2022) (Table 3). In recent years, studies on EVs isolated from different cell sources have demonstrated their potent regenerative potential and anti-inflammatory effects in tendinopathies (Rhatomy et al., 2020). Physiologically, the restoration of tendon homeostasis initiates from the presence of resident TSPCs which are triggered by the tissue injury and release of exosomes that balance tendon ECM synthesis and degradation (Wang et al., 2019). On this basis, TSPC-derived exosomes can be isolated *in vitro* and used as a possible reparative therapeutic strategy for tendon damage. However, little is known about the interaction between TSPC-derived EVs and the target tissue, therefore it is premature to assess possible therapeutic implications. EVs isolated from ASCs or BMSCs are much more studied and used for the treatment of tendinopathies because of their easier and wider accessibility. The literature shows they are effective in immune regulation, in attenuating tissue inflammation and in improving tissue regeneration preclinical models of tendon pathology.

**TABLE 3:** Immunomodulatory and reparative actions of MSC EVs in preclinical tendinopathy models.

References	Tendon injury	Number, age, and gender of animals	Therapeutic group	Control group	Follow-up	Therapeutic effect	Outcomes
Shi et al. (2019)	Patellar tendon defect rat model	Forty-eight rats	Allogeneic BMSC-EVs (25 µg EVs)	Non-treated	4 weeks	Benefit	BMSC-EVs improve the histological appearance of treated tendons and increased the expression of genes related to tissue matrix formation and tenogenic differentiation
			Fibrin				
Wang et al. (2019)	Collagenase-induced Achilles tendinopathy rat model	Eighteen 8 weeks-old male rats	Allogeneic TSPC-EVs (1 mL EVs from 1000 mL TSPC conditioned medium)	Saline	5 weeks	Benefit	Both TSPCs and TSPC-EVs injections significantly decreased matrix metalloproteinases (MMP)-3 expression, increased expression of tissue inhibitor of metalloproteinase-3 (TIMP-3) and Col-1a1, and increased biomechanical properties of the ultimate stress and maximum loading
			Allogeneic TSPCs (1 × 10^6^ cells)				Histological score system showed that scores of TSPCs or TSPC-EVs groups were significantly higher than that of PBS injury group (*p* < .0001)
Shen et al. (2020)	Achilles tendon injury mouse model	Thirty-two 3–4 months-old NF-κB-GFP-luciferase mice	Allogeneic ASC-EVs seeded on collagen sheet (5∼6 × 10^9^ EVs)	Collagen sheet	1 week	Limited Benefit (for naive ASC-EVs)	EVs from IFNγ-primed but not naïve ASCs attenuated the early inflammatory reaction following injury *via* modulation of the macrophage inflammatory response
							IFNγ-primed ASC-EVs are more effective in suppressing NF-κB activation in macrophages (*p* = .006) compared to control
							Compared with control repairs, primed ASC-EVs further reduced the rate of post-repair tendon gap formation and rupture (*p* = .033) and facilitated collagen formation (*p* < .001) at the injury site
							ASC-EVs administration improved histological characteristics and biomechanical strength of the tissue
Li et al. (2020)	Achilles tendon injury rat model	Thirty-three adult male rats	Human UCMSC-EVs (200 μg EVs)	Saline	3 weeks	Benefit	Hydroxycamptothecin-primed UCMSC-EVs show high anti-adhesion potential for the treatment of tendon injury by provoking the expression of endoplasmic reticulum stress (ERS)-associated proteins in fibroblasts
			Human hydroxycamptothecin-primed UCMSC-EVs (200 μg EVs)				
Zhang et al. (2020)	Achilles tendon injury rat model	Sixty-two adult rats	Allogeneic TSPC-EVs with gelatin methacryloyl (200 μg EVs)	Non-treated	8 weeks	Benefit	TSPC-EVs significantly suppress inflammation and apoptosis 1 week after surgery
			Gelatin methacryloyl				At later time points, the tissue treated with TSPC-EVs showed improved collagen fiber alignment, unlike the control group
Yu et al. (2020)	Patellar tendon defect rat model	Fifty-two adult male rats	Allogeneic BMSC-EVs with fibrin glue (20 μg EVs)	Fibrin glue	4 weeks	Benefit	BMSC-EVs with fibrin glue significantly improved histological scores (7 ± 2 vs. 10 ± 2, *p* = .021), increased the expression of Mohawk, Tenomodulin and Collagen I compared to control
							BMSC-EVs with fibrin glue significantly improved the biomechanical properties of the neo-formed tendon, and promoted the proliferation of resident TSPCs compared to the control
							The stress at failure of the healing tendons and modulus were 1.84-fold (13 ± 5 MPa vs. 7 ± 2 MPa, *p* = .034) and 1.86-fold (39 ± 8 MPa vs. 21 ± 4 MPa, *p* = p.012) higher in BMSC-EVs with fibrin glue group compared to control, respectively
Wang et al. (2020)	Chronic rotator cuff tear rabbit model	Thirty-five 4 months-old male rabbits	Human ASC-EVs (10^11^ EVs)	Saline	18 weeks	Benefit	ASC-EVs resulted in inhibition of fat infiltration and fibrocartilage formation, and an increase of the biomechanical strength
							The mean ultimate load to failure of the ASC-EVs group (132.7 ± 10.3 N) was significantly greater than that in the saline group (96.0 ± 9.8 N) (*p* < .001)
Wang et al. (2021)	Chronic rotator cuff mouse model	Eighty-one wild-type C57BL/6 male mice	Human ASC-EVs (10^11^ EVs)	Saline	4 weeks	Benefit	Local administration mitigates the inflammatory response *via* macrophage polarization towards anti-inflammatory (M2) phenotype, and thus restoring the physiological M1/M2 balance
							Treadmill overuse significantly increased the M2 polarization rate (CD206+/F4/80+) from 4.78% ± 2.07% in the normal group to 30.86% ± 7.04% in the ASC-EVs group (*p* < .05)

### 4.1 MSC-derived EVs immunomodulatory actions in preclinical models

In a mouse Achilles tendon injury model, ASC-EVs local transplantation with collagen sheets attenuated the early inflammatory reaction following injury *via* modulation of the macrophage inflammatory response (Shen et al., 2020). Furthermore, in a chronic rotator cuff mouse model Wang et al. showed that exosome local administration mitigates the inflammatory response *via* macrophage polarization towards anti-inflammatory (M2) phenotype, and thus restoring the physiological M1/M2 balance (Wang et al., 2021). Collectively, these anti-inflammatory effects are thought to be mediated by blocking macrophage NF-kB activity (Shen et al., 2020). Although less investigated, other types of MSCs have also been considered as potential sources of secretomes and extracellular vesicles. Considering the secretome obtained from human amniotic membrane-derived mesenchymal stromal cells (AMSCs), an extensive characterization of secreted factors and a targeted analysis based on miRNAs related to tendinopathies showed that the most abundant EV-miRNAs are teno-protective while capable of inducing polarization of M2 macrophages, inhibiting inflammatory T lymphocytes, and promoting Treg (Ragni et al., 2021). Another study investigated the effects of EVs obtained from Umbilical cord derived MSCs (UCMSC-EVs) that demonstrated beneficial effects in a rat model of Achilles tendon injury (Li et al., 2020).

In order to enhance further the effects of cell-free therapeutic approaches, *in vitro* priming can increase the release and enrich the content of the secreted EVs in the desired molecules/factors. EVs obtained from primed cells *in vitro* generally show more potent effects than those obtained from their non-induced counterparts (Shen et al., 2020). Specifically, *in vivo* MSCs exposed to an inflammatory milieu exhibit immunomodulatory/anti-inflammatory and anti-fibrotic effects, exerted in part through the release of paracrine mediators (Uccelli and Rosbo, 2015; Galipeau et al., 2016). These molecular mechanisms are enhanced with prior exposure to immune cells and/or environments rich in IFNγ, TNFα and IL-1α (Renner et al., 2009; Krampera, 2011; Kadle et al., 2018; Kouroupis et al., 2019b; Kouroupis et al., 2020). On this basis, EVs isolated from IFN-γ induced ASCs more efficiently than naïve ones, reduce the rate of post-repair tendon gap formation and rupture, and increase collagen formation at the injury site (Shen et al., 2020).

### 4.2 MSC-derived EVs reparative actions in preclinical models

In a collagenase-induced rat Achilles tendinopathy model, Wang et al. showed that upon TSPC-derived exosome intra-tendinous administration, tendon repair was promoted both histologically and biomechanically with effects comparable to TSPCs (Wang et al., 2019). In another *in vivo* study, a model of Achilles tendinopathy was established in Sprague Dawley rats and the results demonstrated that exosomes obtained from TSPCs could significantly suppress inflammation and apoptosis 1 week after surgery. At later time points, the tissue treated with TSPC-derived exosomes showed improved collagen fiber alignment, unlike the control group (Zhang et al., 2020).

BMSC-EVs also demonstrate therapeutic efficacy in tendinopathies. In a pre-clinical model of patellar tendon defects in Sprague-Dawley rats, treatment with BMSC-EVs improved the histological appearance of treated tendons and increased the expression of genes related to tissue matrix formation and tenogenic differentiation (Shi et al., 2019). BMSC-derived exosomes embedded in fibrin scaffold and injected into the defect area of rat patellar tendons significantly improved histological scores, increased the expression of Mohawk, Tenomodulin and Collagen I, improved the biomechanical properties of the neo-formed tendon, and promoted the proliferation of resident TSPCs (Yu et al., 2020).

Recent studies showed the tendon reparative properties of ASC-EVs. When administered in a rabbit model of chronic tears, inhibition of fat infiltration and fibrocartilage formation was observed together with an increase of the biomechanical strength (Wang et al., 2020). EV reparative effects were evident in a mouse Achilles tendon injury and repair model too as their administration facilitated tendon matrix regeneration with the increase of *COL1A1* and *COL3A1* and the attenuation of *MMP1* expression. Moreover, EVs administration improved histological characteristics and biomechanical strength of the tissue. In fact, the EV-treated tendons exhibited a higher percentage of collagen staining at the site of tendon injury with an even greater effect when ASCs were primed with inflammatory stimuli before EV collection (Shen et al., 2020).

In addition to a whole series of advantages already observed in terms of effectiveness, cell-free applications have generated enormous interest as they comply with regulatory requirements and minimize concerns related to safety if used in patients (L et al., 2019). Unlike MSCs, MSC-derived exosomes are more stable and controllable entities, with fewer safety risks associated with cell injection, such as the risk of microvascular occlusion (Moghadasi S et al., 2021). In contrast to other pathologies where the use of EVs is at a more advanced stage with registered clinical trials evaluating their safety and efficacy (Moghadasi et al., 2021), their clinical application for tendinopathies is still in the nascent stages. Accordingly, the scientific community is striving to identify the most efficient protocol of isolating and applying EVs for tendon pathologies treatment by conducting several *in vitro* and preclinical studies (Lui, 2021; Ragni et al., 2022). Additionally, large-scale randomized controlled trials are needed to confirm no adverse effects and most importantly long-term functional improvements. Specifically, long-term adverse effects related to immunologic reactions should be thoroughly investigated. These studies will also clarify and standardize technical issues related to mode of MSC-derived EVs infusion, infusion dosage, and number of infusions for better long-term functional improvements. On this basis, further large-scale and long-term studies will help to overcome current hurdles for MSC-derived EVs clinical translation in tendinopathies.

## 5 Conclusion

**FIGURE 2:**
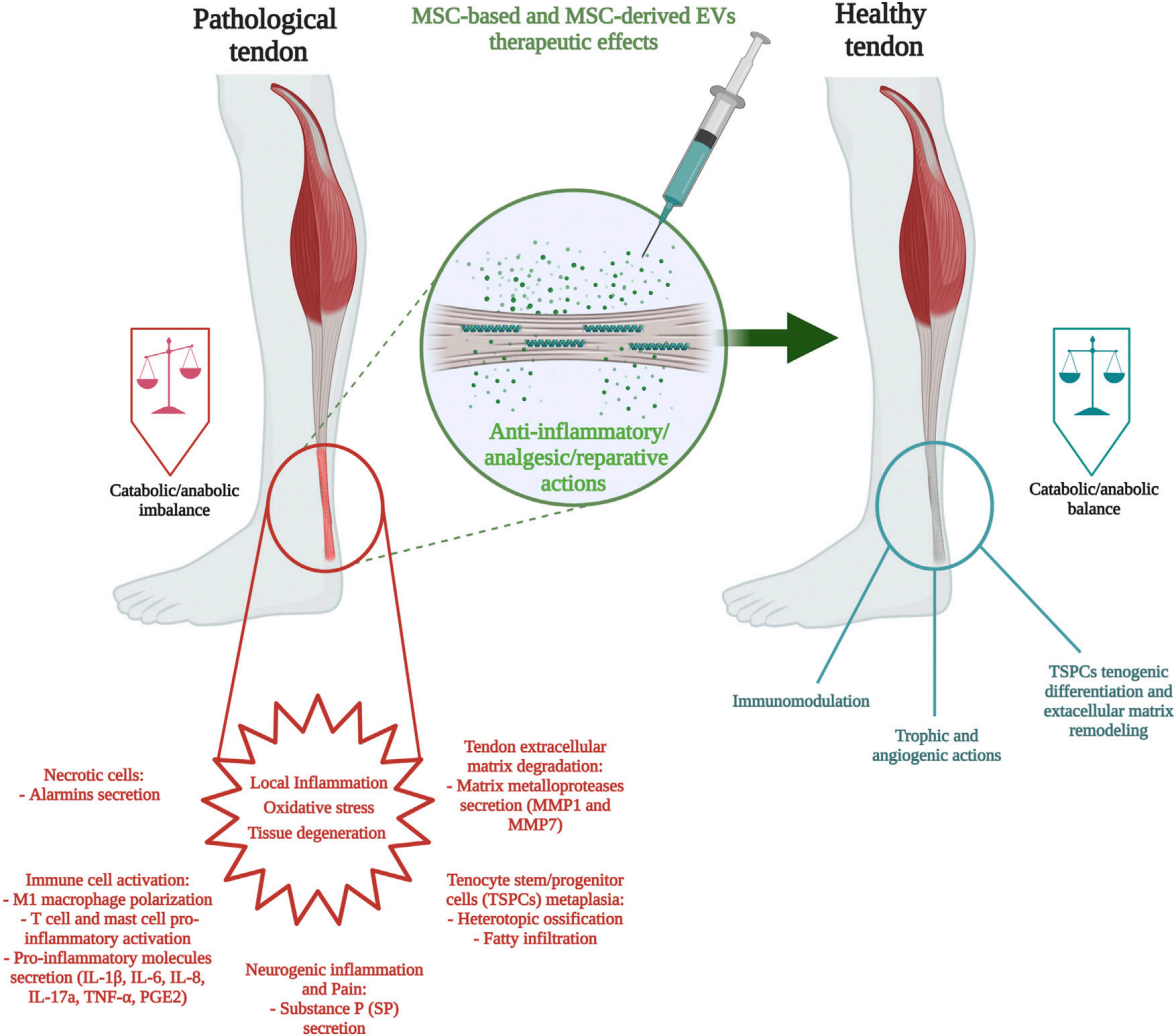
Components of tendon pathology and MSC-based/MSC-derived extracellular vesicle therapeutic approaches.

Overall, MSC-based and MSC-derived products (secretome and extracellular vesicles) show promise as alternative treatment approaches for tendon pathologies. Specifically, preclinical and several clinical studies have demonstrated that the catabolic/anabolic imbalance inititated by oxidative stress, local inflammation, and tissue degeneration can be restored to a catabolic/anabolic state *via* MSC anti-inflammatory/analgesic/reparative actions (Figure 2). On this basis, the adaptation of regulatory-compliant reproducible methods for MSCs and MSCs secretome production, and their comparative evaluation to current conventional treatments in preclinical and clinical studies would contribute to their establishment as safe and effective therapies for tendon pathologies.
